# On the semantic representation of risk

**DOI:** 10.1126/sciadv.abm1883

**Published:** 2022-07-08

**Authors:** Dirk U. Wulff, Rui Mata

**Affiliations:** 1 University of Basel, Basel, Switzerland.; 2 Max Planck Institute for Human Development, Berlin, Germany.

## Abstract

What are the defining features of lay people’s semantic representation of risk? We contribute to mapping the semantics of risk based on word associations to provide insight into both universal and individual differences in the representation of risk. Specifically, we introduce a mini-snowball word association paradigm and use the tools of network and sentiment analysis to characterize the semantics of risk. We find that association-based representations not only corroborate but also extend those extracted from past survey- and text-based approaches. Crucially, we find that the semantics of risk show universal properties and individual and group differences. Most notably, while semantic clusters generalize across languages, their frequency varies systematically across demographic groups, with older and female respondents showing more negative connotations and mentioning more often certain types of activities (e.g., recreational activities) relative to younger adults and males, respectively. Our work has general implications for the measurement of risk-related constructs by suggesting that “risk” can mean different things to different individuals.

## Teaser

Free associations reveal commonalities and differences in the semantic representation of risk across languages, age, and gender.

## INTRODUCTION

Today’s societies require individuals to navigate an increasing number of social and technological risks (*1*, *2*). But how do individuals think about risk? Different disciplines have provided several conceptions that are not fully compatible (*3*–*5*)—a fact that may have contributed to conceptual and empirical confusion regarding the construct in the cognitive and behavioral sciences [e.g., (*6*)]. For example, while some have emphasized risk as being a statistical concept [i.e., variance; (*3*)], others have emphasized its subjective character and multiple psychological dimensions [e.g., (*7*)]. Crucially, despite a long-standing interest in understanding individual differences in risk-related constructs [e.g., (*8*, *9*)], comparatively little attention has been given to the fact that the meaning of risk may differ between individuals as a function of different goals and life experiences [e.g., (*10*)]. In our work, we propose a novel method to uncover the semantic representation of risk and assess potential individual- and group-related differences in this concept central to the behavioral sciences.

### Past approaches to the psychology of risk

There have been several approaches to understanding the main psychological dimensions associated with risk and risk-related constructs, such as risk perception or risk taking. One prominent survey-based approach uses dimensionality reduction to capture the dimensions of different activities and technologies based on people’s ratings of such activities on several aspects [e.g., perceived risk, perceived benefits, and controllability; (*11*)]. This work has concluded that different technological risks and activities, from nuclear energy to smoking, can be mapped onto a psychological space composed of (at least) two dimensions, often termed dread and uncertainty. A second survey-based approach also relies on dimensionality reduction to capture the dimensions of the propensity to engage in various risky behaviors covering domains of life for which individuals are thought to have relatively independent beliefs (*9*). This has become a leading approach to measuring individuals’ risk attitudes and has concluded that individuals have different psychological representations of the benefits and risks of each life domain, such as the recreational, occupational, financial, or social domains (*9*). A third approach has mapped the semantic representation of risk from text corpora by tabulating the most frequent definitions in lexical sources, such as dictionaries and thesauri (*4*), or mapping the semantic networks of word associates from other text sources, such as Wikipedia, to obtain a multidimensional space (*2*). These text-based approaches have typically revealed more complex semantic representations that can involve dozens of different components or “semantic fields” [cf. (*4*)].

The approaches discussed above have provided considerable insight into the semantic representation of risk but are not without limitations. First, those past survey-based approaches that relied on experimenter-generated aspects or behaviors [e.g., risky technologies and behaviors; (*9*, *11*)] cannot guarantee that these exhaustively capture all aspects or dimensions of the semantic representation of risk. For example, there have been recent calls to expand the psychology of risk to examine positive risk taking that has been rather neglected in past research into the topic [e.g., (*12*)].

Second, lexical approaches that relied on a naturalistic and broad set of lexical sources are more likely to have identified a full range of aspects or dimensions, but their reliance on aggregate information does not allow investigation of the role of individual and group differences in the semantic representation of risk [e.g., (*2*, *4*)]. A number of past findings, however, suggest that the semantic representation of risk differs across groups and individuals. For example, past work suggests age and gender differences in risk-related constructs, such as risk perception [e.g., (*13*–*15*)] and risk taking [e.g., (*10*, *16*–*18*)], which may be anchored in different semantic representations of risk.

Here, we introduce a novel approach that aims to directly uncover the semantics of risk in a data-driven manner while assessing potential differences between demographic groups (e.g., younger versus older and males versus females). Doing so promises to help uncover to what extent these individual differences matter for our assessment and prediction of risk-related behaviors.

### The promise of word associations to mapping the semantics of risk

In our work, we introduce a novel approach to uncovering the semantics of risk that uses word associations. Word associations are amenable to the investigation of individual differences and arguably provide more direct access to individual subjective meaning than both survey-based approaches reliant on experimenter-generated stimuli and text-based approaches while retaining the breadth of the latter approaches (*19*, *20*). Past research has found semantic representations extracted from word associations to be more predictive of human judgments and behavior than those extracted from co-occurrences of words in text corpora (*19*, *21*, *22*). This is likely because word association reflects other factors beyond semantic relationships, such as pragmatic communication rules (*19*). Word associations thus promise to enrich existing perspectives on the concept of risk.

**Fig. 1.**
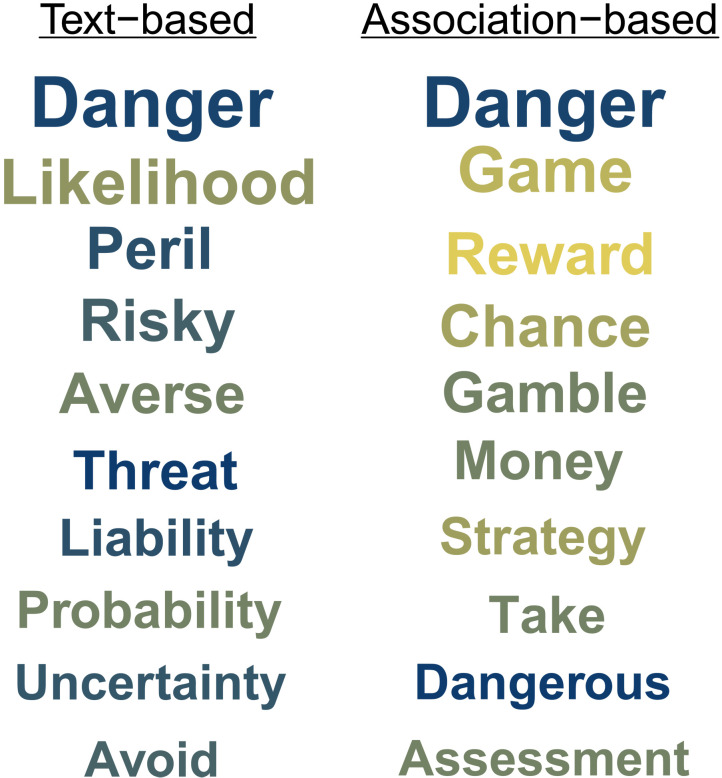
Comparison of text-based and free association–based approaches to the semantics of risk. Left column shows the 10 words with the highest cosine similarity to the word “risk” based on the fastText pretrained Word2Vec representation from Mikolov *et al*. (*23*). Right column shows the 10 most frequent associations in the English Small World of Words (SWOW) free association database from De Deyne *et al*. (*24*). The words’ size reflects cosine and retrieval frequency, respectively. The words’ color reflects sentiment based on the SentiWordNet sentiment dictionary (*57*).

A preliminary example will help make the case for a word association approach relative to text-based approaches in uncovering the psychology of risk. We compared text-based and word association-based approaches to the semantics of risk by analyzing words with the highest cosine similarity to the word “risk” based on text-based representations estimated from large text corpora (*23*) and association-based representations estimated from a large-scale citizen-science project involving thousands of free associations (*24*) and compared their retrieval frequency and sentiment. Figure 1 shows that there is relatively little overlap in the top 10 associates of “risk” in existing text-based and association-based representations. Crucially, associations derived from word associations appear to have more diverse meaning and sentiment than those derived from text whereas text-based representations mainly focus on negative consequences, and uncertainty association-based representations also feature positive aspects such as “reward.” This suggests that word associations may shed light on aspects of the semantic representation of risk that are less accessible through other approaches.

### The current study

We propose using a mini-snowball, word association method to map the semantic representation of risk directly from individuals’ associations and assess relevant individual and group differences. Specifically, our word association task asked participants to name five associates of the word “risk” and five associates of each of the initially generated risk associates (for a total of 30 associates per participant). We collected responses from a sample of 1205 German individuals using a nationally representative online sample from a market research firm. The sample was balanced in terms of age and gender by using sampling quotas for six age groups (age range = 17 to 87, *M* = 47.7, and SD = 16.6) and gender (50% female).

Our study answers four main questions. The first question concerns the extent to which the components of risk stemming from our approach qualitatively match the different dimensions and domains that have been typically identified in past research [e.g., (*4*, *7*, *9*)]. Second, we address the extent to which semantic representations of risk generalize across languages and therefore have some universal character that captures general ways in which humans think about risk. Third, concerning group differences, our analysis aims to describe potential age and gender differences in the representation of risk, including the types or domains (e.g., recreational) and other characteristics (e.g., sentiment) of risk associates. Past work has identified clear and robust patterns of age and gender differences in risk-related constructs [e.g., (*17*)], and our study extends such work by considering whether similar group differences are found directly in the semantic representation of risk. Fourth and last, we ask whether individual differences in the semantic representation of risk can be used to better understand and predict individuals’ risk taking, thus contributing to the goal of assessing the power of semantic representations in predicting individual and group differences in risk-related behaviors.

The remainder of our article is structured as follows: First, we present a general semantic network of risk created from the responses of all participants and characterize its components. Second, we report our efforts to compare the semantic network of risk obtained from our mini-snowball approach for the German language to data from two additional languages, Dutch and English. Third, we present results on age and gender differences in the semantic representation of risk at the level of risk components and individual words. Last, we present results on the link between people’s semantic representations and self-reported risk-taking propensity. Note that, although English terms are presented in text and figures, all analyses were run using the original languages (i.e., German and Dutch).

## RESULTS

### The semantic network of risk

**Fig. 2.**
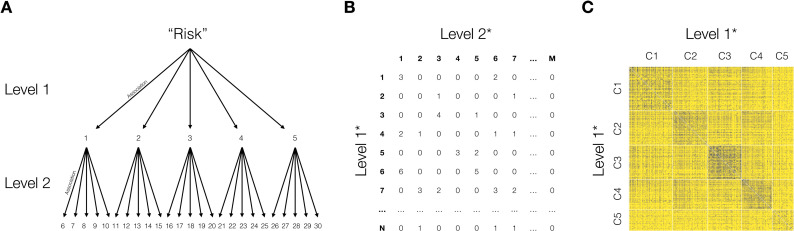
Construction of the semantic network of risk. (**A**) Mini-snowball word association approach used to generate level 1 and level 2 associates of the word “risk” (numbers represent each of the 30 word associates provided by each respondent). (**B**) Level 1 by level 2 co-occurrence matrix, which served as the basis for determining the relatedness among the level 1 responses (numbers represent unique associates). (**C**) Relatedness matrix between level 1 associates, determined by calculating the Jaccard similarity between rows in the co-occurrence matrix shown in (B) and ordered according to a clustering algorithm (see main text for details; C, component). The asterisk (*) indicates that only the subset of level 1 associates are included that were produced at least three times across all participants.

To identify the semantic components of risk, we constructed a general semantic network of the immediate associates of risk (level 1) using the data of all participants. Figure 2 depicts our approach [for related approaches, see (*25*, *26*)]. In a first step, we characterized each level 1 response that occurred at least three times (see Fig. 2A) by means of the frequency distribution of level 2 responses that they elicited (see Fig. 2B). In a second step, we determined the relatedness of level 1 responses by calculating a weighted Jaccard similarity between them (*27*), which has been found to perform well relative to other similarity measures in clustering approaches [e.g., (*28*–*30*)]. In a third step, we represented the similarity matrix of level 1 responses as a weighted network and extracted the components of risk using the Louvain modularity algorithm (*31*), which has been found to compare favorably to other modularity and clustering algorithms [e.g., (*32*–*35*)]. One attractive feature of modularity detection algorithms, such as the Louvain algorithm, is that they also identify an optimal number of clusters with respect to maximizing modularity. It is important to note, however, that there exists no general, a priori correct clustering algorithm for such problems (*36*, *37*).

**Fig. 3.**
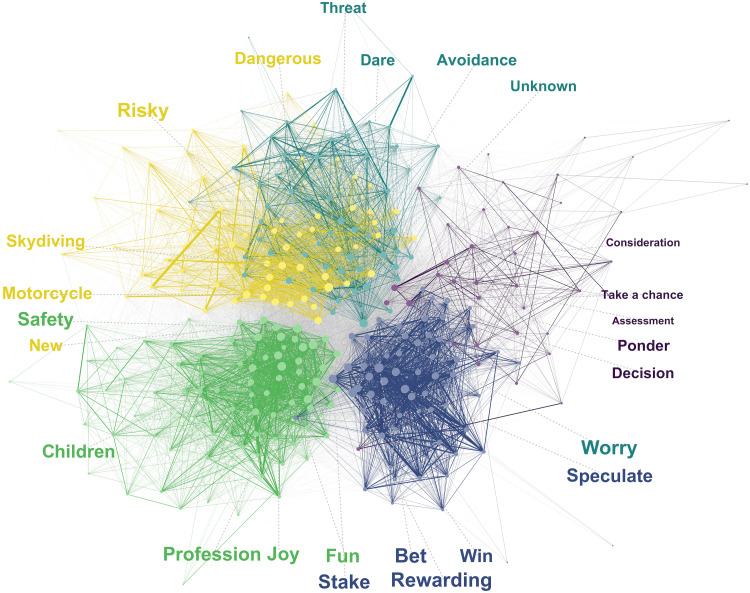
The semantic network of risk. Nodes represent the 307 distinct level 1 associates sized according to their importance for the network (PageRank). Edges represent the relatedness between nodes sized by the magnitude of the Jaccard index. Colors represent the five components identified by the Louvain algorithm (*31*). For each component, the five most important words are shown sized by importance. Gray lines in the background represent between-component edges. The layout of the nodes was determined using the Fruchterman-Reingold algorithm (*58*) including a minor edge weight bias for within-component edges to increase visual separation of the components.

**Fig. 4.**
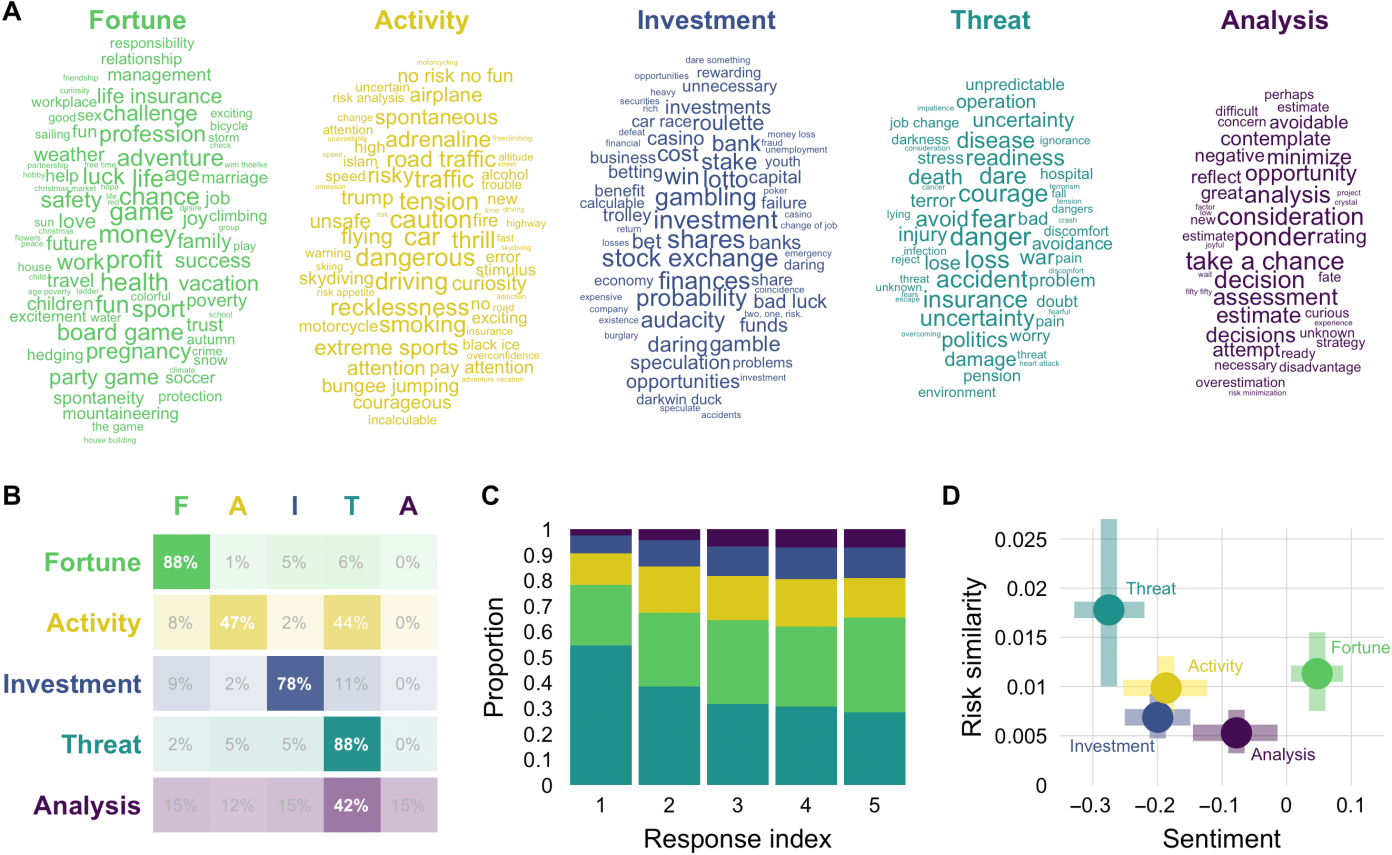
The semantic components of risk. (**A**) Word clouds of all words contained in the respective component, with the size reflecting the retrieval frequency rank of the word. (**B**) Cluster stability. (**C**) Retrieval proportions for each of the five components from the first up to the fifth associate of risk. (**D**) Average sentiment and proximity to risk for each of the five components. Error bars indicate bootstrapped 95% confidence intervals.

The semantic network of risk was found to consist of five components. Figure 3 displays the network and its five components along with the five most important words of each component with respect to their PageRank centrality in the entire network. Figure 4A further illustrates the network by showing word clouds for each component, with words scaled by their frequency of occurrence. The largest component encompassing the most distinct words (*n* = 82 of 307), which we labeled as Fortune, contained words pertaining to positive outcomes of risk, such as “money,” “profit,” and “fun,” as well as many aspects pertaining to life in general, where risks may be involved, such as “health,” “occupation,” and “children.” The second largest component (*n* = 64), labeled as Investment, contained words pertaining to financial investment, such as “shares,” “stock exchange,” “stake,” and “gambling.” An equally large third component (*n* = 64), labeled Activity, contained words pertaining to various kinds of activities and activity-related words, such as “driving,” “car,” “motorcycle,” “smoking,” and “caution.” The fourth largest component (*n* = 57), labeled as Threat, contained words pertaining to the negative consequences of risk, such as “danger,” “fear,” “loss,” “accident,” and “dare.” Last, the smallest component (*n* = 40), labeled Analysis, contained words pertaining to the deliberate aspects of taking risks, such as “ponder,” “decision,” “trade-off,” and “assessment.”

The five components of risk varied in their distinctiveness according to a clustering stability analysis (*37*); see also the Supplementary Materials. We bootstrapped level 2 responses and assessed for each level 1 response how frequently it shared clusters with words from each of the components. Figure 4B depicts the proportion of modal cluster assignments for the words in each component; that is, the component that supplied most of the fellow cluster members for each word (while controlling for component size). This shows that most words in the components Fortune (88%), Threat (88%), and Investment (78%) were primarily found to share clusters with words from their original component, suggesting high distinctiveness and robustness for these components. Words in the components Activity (47%) and Analysis (42%) shared clusters with words from their original component much less often, suggesting lower levels of distinctiveness. Notably, almost half of the words of the component Activity preferably shared clusters with words of the component Threat, suggesting a strong link between these two components.

The components further varied strongly in retrieval frequency. Note that retrieval frequency is different from component size, which is primarily driven by response diversity, not frequency. Overall, words of the component Threat accounted for 37.5% of all level 1 responses and 54.5% of first responses (see Fig. 4C). From this component, “danger” is by far the most frequent response, produced by 43.4% of participants and by 27.9% at the first position. Words of the component Fortune accounted for 30.4% of level 1 responses and were the most frequent responses at position four and five. From this component, “money” was the most frequent word, produced by 12.9% of participants. The three remaining components, Activity, Investment, and Analysis, accounted for 16.2, 10.5, and 5.4% of responses, respectively. The most frequently retrieved words from these components were “car” (1.2%), “shares” (1.2%), and “ponder” (0.5%).

Last, as foreshadowed by our preliminary comparison of text- and association-based representations (see Fig. 1), the components varied strongly in their semantic and affective content. Figure 4D characterizes the components in terms of the components’ similarity to the concept of risk, determined on the basis of the Jaccard similarity matrix and sentiment, with the latter determined using the SentiWS dictionary [see Materials and Methods; (*38*)]. Most notably, the two components most similar to the concept of risk, Threat and Fortune, are at polar ends of the sentiment spectrum, with Threat being the most negative and Fortune being the most positive component. The three remaining components, Activity, Investment, and Analysis, are less centrally related to the concept of risk and have moderately negative sentiment.

In summary, the general network of risk revealed five thematically distinct components. A component labeled as Threat, containing mostly negative consequences of taking risks, emerged as the most important in terms of retrieval frequency and similarity to the concept of risk. The second most important component, labeled Fortune, consisted of positive consequences and other life-related, mainly positive associates of risk. In addition to these two focal components, three other components capture risky situations, such as financial investment (Investment) and risky leisure activities (Activity), as well as higher-level considerations associated with deliberating about risks (Analysis). All in all, these results support the idea that the risk concept is multifaceted and associated with both negatively and positively valenced components.

### The semantic network of risk across languages

An important question that emerges from the above analysis is whether the semantic representation of risk that we identified in the German language generalizes to other languages. To investigate this issue, we conducted a comparison of the semantic network of risk that we obtained using our snowball approach to those of two other languages, Dutch and English. For this purpose, we used data from the Dutch and English Small World of Words (SWOW) projects (*24*, *39*). The SWOW project aims to map semantic representations for multiple languages using a massive word association task adopting a citizen-science approach. Our approach involved identifying all pairs of words in the semantic network of risk that we identified from our mini-snowball approach for the German language to predict the similarity for the same pairs from the Dutch and English languages from the SWOW data. The German network was composed of 307 terms, and the SWOW projects provided estimates for the majority of these, namely, 244 (79%) and 253 (82%) terms for the Dutch and English languages, respectively.

**Fig. 5.**
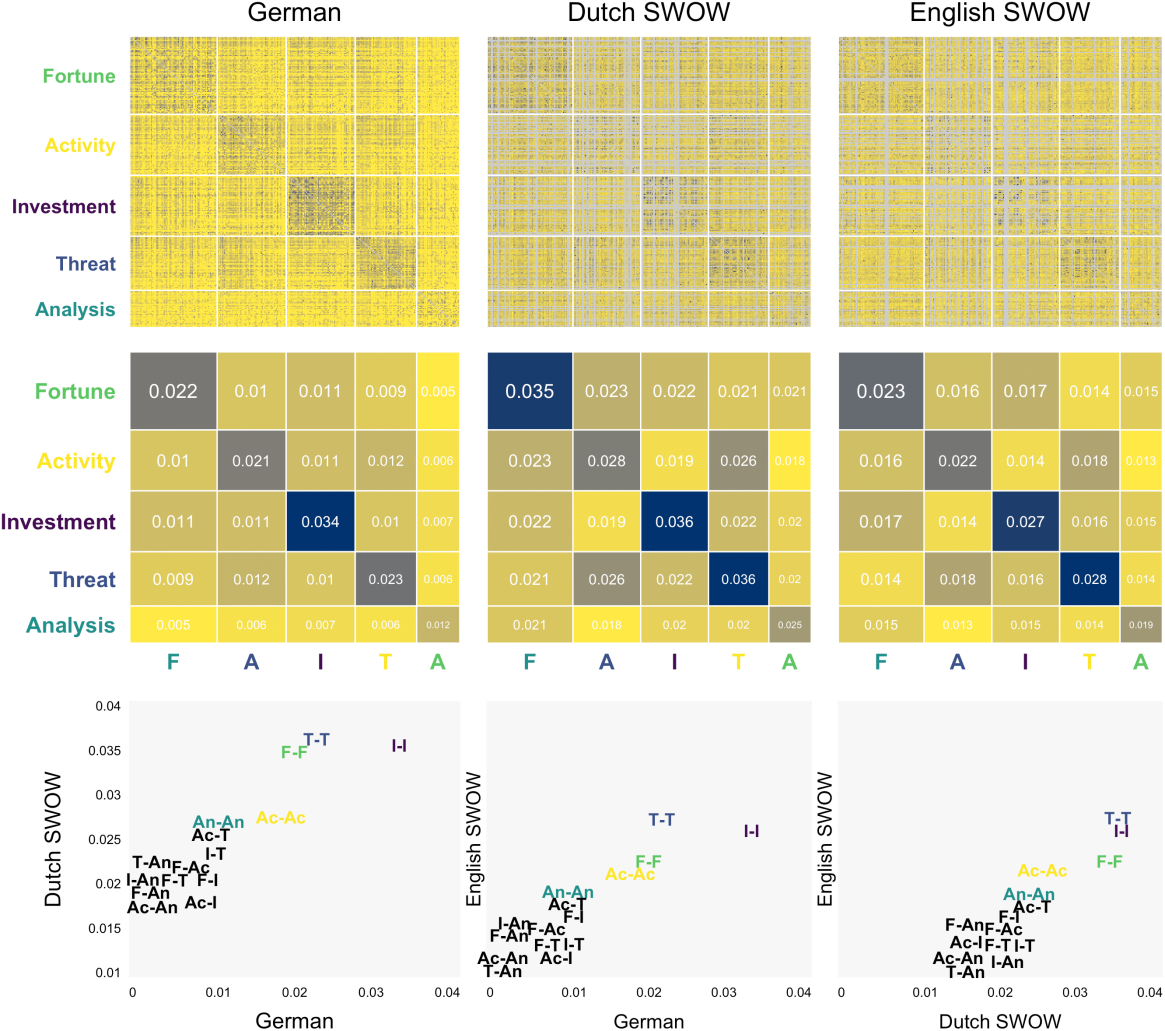
The semantic network of risk in other languages. The top row shows the Jaccard similarities for all pairs of words in the semantic network of risk based on the current data and data from the Dutch and English SWOW projects. The latter two provide estimates for pairs involving 244 and 253 of the 307 terms in the network of risk. Missing terms in the Dutch and English SWOW are indicated by gray lines. The middle row shows the average similarities within and between risk components for all three languages. The bottom row illustrates the correlation between component similarities for pairs of languages.

Figure 5 presents the main results of this analysis, including (i) the Jaccard similarities for all pairs of terms in the semantic network of risk for the three languages, (ii) the mean similarities within and across the five clusters that we had identified (i.e., Fortune, Activity, Investment, Threat, and Analysis), and (iii) the overall correlation between the similarity between pairs across each of the languages (i.e., German-Dutch, German-English, and Dutch-English).

Two main conclusions can be drawn from these results. First and foremost, the results suggest that the clustering identified for the German language largely generalizes across languages, with the similarities within clusters showing larger similarity, on average, relative to the similarities across clusters. Second, the overall similarity between pairs is quite robust across languages showing correlations between 0.92 and 0.97.

We further analyzed retrieval frequencies for associates to the word “risk” in these languages. In Dutch, most responses belonged to the component Threat (48.6%), followed by Activity (23.1%), Fortune (15.6%), Finance (10.4%), and Analysis (2.3%). In English, most responses belonged to the component Fortune (37.9%), followed by Threat (29.6%), Finance (14.8%), Analysis (9.5%), and Activity (8.2%). Compared to German, Dutch-speaking respondents consequently placed an even greater emphasis on Threat and Activity and less emphasis on Fortune, whereas English-speaking respondents placed greater emphasis on Fortune and Finance and less emphasis on Threat. Nevertheless, retrieval proportions for the risk components were quite similar across languages, with overall strong correlations between the retrieval proportions between German and Dutch [correlation coefficient (*r*) = 0.82] and German and English (*r* = 0.84).

Last, we also evaluated the components’ sentiment and risk similarity using the Dutch and English data (see the Supplementary Materials). Consistent with the results in German (see Fig. 4D), Threat and Fortune emerged as the most negative and most positive components, respectively, in both Dutch and English. Moreover, Threat was found to have high risk similarity, while Analysis was found to have low risk similarity. However, there were also small but notable inconsistencies between languages. In particular, Investment, which had a low risk similarity in the German data, showed equivalent risk similarity than Threat for both Dutch and English. These results point out that finding major similarities in the representation of risk across languages does not preclude some differences for specific components, potentially indicating some cross-cultural differences in the representation of risk.

All in all, these results suggest that the structure of the semantic network of risk shows considerable similarities across languages and may be a general property of how individuals understand risk. One should note, however, that large commonalities in the semantic representation of risk across languages do not imply perfect similarity across individuals or groups within that language or culture. In the following, we investigate potential systematic differences across groups.

### Group differences in the semantic representation of risk: The role of age and gender

Our association-based approach to mapping the semantics of risk promises to help characterize both universal and group-specific differences in the psychology of risk. In what follows, we demonstrate the possibility of characterizing group differences by exploring differences in the semantic representation of risk as a function of individuals’ age and gender.

**Fig. 6.**
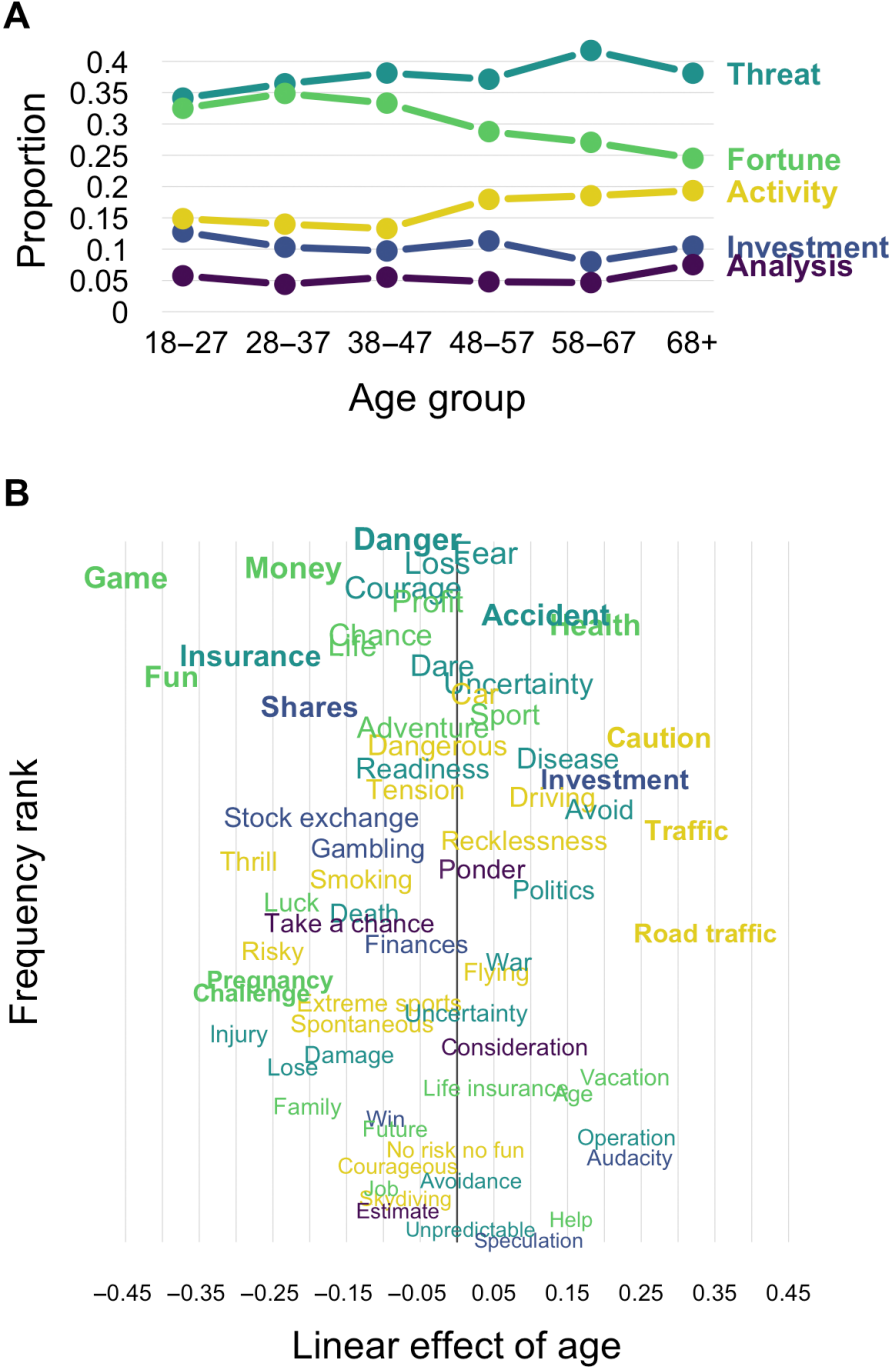
The semantic representation of risk across age. (**A)** Proportion of retrievals falling into each of the five components across the different age groups. (**B**) Word-level changes in retrieval frequencies of words in terms of the linear effect of age. Specifically, the panel shows the unstandardized effect of a predictor coding age group predicting the log-scaled relative retrieval frequencies. Words in bold showed a significant age difference in retrieval frequencies at the 0.05 level as determined by a log linear model.

To characterize the semantic representation of risk across age, we analyzed retrieval proportions for each component and level 1 responses across the six age groups in our sample (see Materials and Methods). As can be seen in Fig. 6A, changes in retrieval frequency of components across age were moderate, with the relative order of retrieval proportions of the five components emerging as highly stable across age. Changes across age groups seemed to occur mainly from the transition between age groups of 38 to 47 and 48 to 57, with Threat rising in prominence and Fortune declining. Minor changes also seemed to occur for Activity showing a mild but steady increase with age.

Figure 6B further illustrates the component trends by showing the trajectories of individual, prominent words across the life span. Specifically, the figure shows the linear development across age for each of the 86 words of the network that were retrieved at least once by each of the age groups. Positive linear trends imply that the retrieval proportion increases with age. The illustration reveals systematic changes that cut across the components of risk but clearly correspond to important ecological changes across the life span. For instance, many words more frequently retrieved by older adults center around mobility (“road traffic,” “traffic,” “driving,” and “holiday”) or health (“disease,” “health,” and “operation”). On the other hand, many words more frequently retrieved by younger age groups center on entertainment (“game,” “fun,” “adventure,” “thrills,” and “skydiving”) and financial prospects (“stock exchange,” “money,” “shares,” and “insurance”). Notably, the most frequent associates of “risk,” such as “danger,” “fear,” “accident,” and “loss,” show very small age effects, reinforcing the observation of conceptual stability across age.

**Fig. 7.**
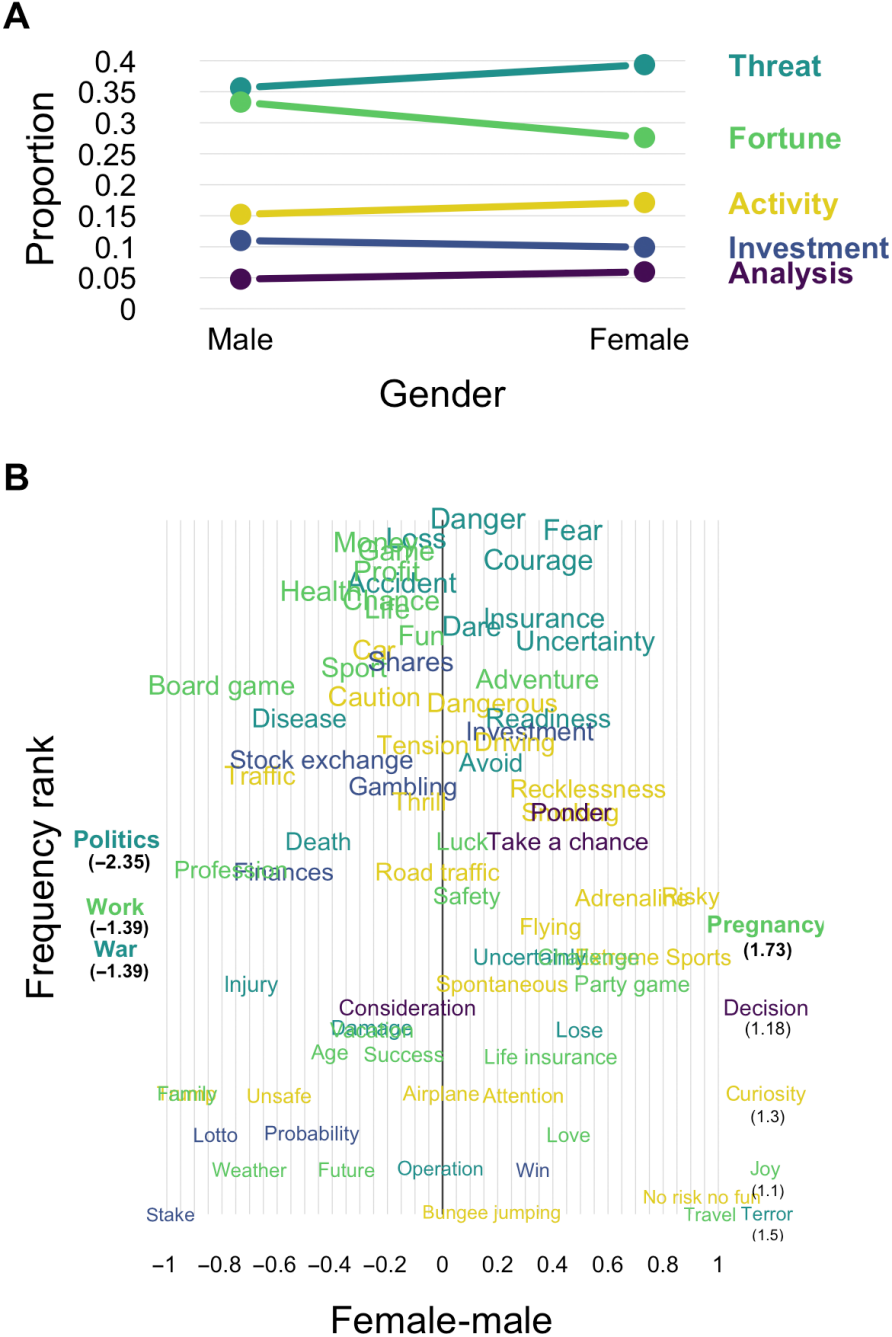
The semantic representation of risk by gender. (**A**) Proportion of retrievals falling into each of the five components across genders. (**B**) Word-level changes in retrieval frequencies of words in terms of the difference of genders. Specifically, the panel shows the difference between the log relative retrieval frequencies. Words in bold showed a significant gender difference in retrieval frequencies at the 0.05 level as determined by a chi-square test for stochastic independence.

Analogous to our analysis of age, we characterized the semantic representation of risk across gender (i.e., males and females) using retrieval frequencies. As can be seen in Fig. 7A, differences in retrieval frequencies were mostly restricted to Threat and Fortune, with females showing a higher proportion for Threat and a lower proportion for Fortune as compared to males. Females also appeared to produce slightly more responses for Activity than males, whereas there were no noticeable differences for Investment or Analysis.

Figure 7B shows the corresponding word-level differences in retrieval frequencies with positive values indicating a higher proportion for females as compared to males. The word-level differences mirrored the component-level differences with many frequent words for Threat (“fear,” “courage,” “uncertainty,” and “danger”) being more frequently produced by females, and many characteristic words for Fortune (“money,” “game,” “chance,” and “profit”) being more frequently produced by males than vice versa. Nevertheless, there also were word-level differences that cut across the components of risk. For instance, the word showing by far the largest difference in the direction of higher proportions for females relative to males was “pregnancy,” whereas the largest differences in the other direction was observed for words pertaining to duty (“policy,” “work,” and “war”), potentially reflecting gender differences in social roles. Notwithstanding these differences, the most frequent associates of risk show comparatively small effects, suggesting relative conceptual stability between males and females.

### Linking individual differences in semantic representations to risk-taking propensity

The previous sections revealed critical group differences in the importance of the components of risk as reflected in retrieval frequencies. Most notably, both older versus younger and female versus male participants tended to produce more associates belonging to the overwhelmingly negatively valenced component Threat and fewer associates belonging to the more positively valenced component Fortune. Could the associative frequencies help understand individual differences in risk taking, over and beyond established effects of age and gender?

**Fig. 8.**
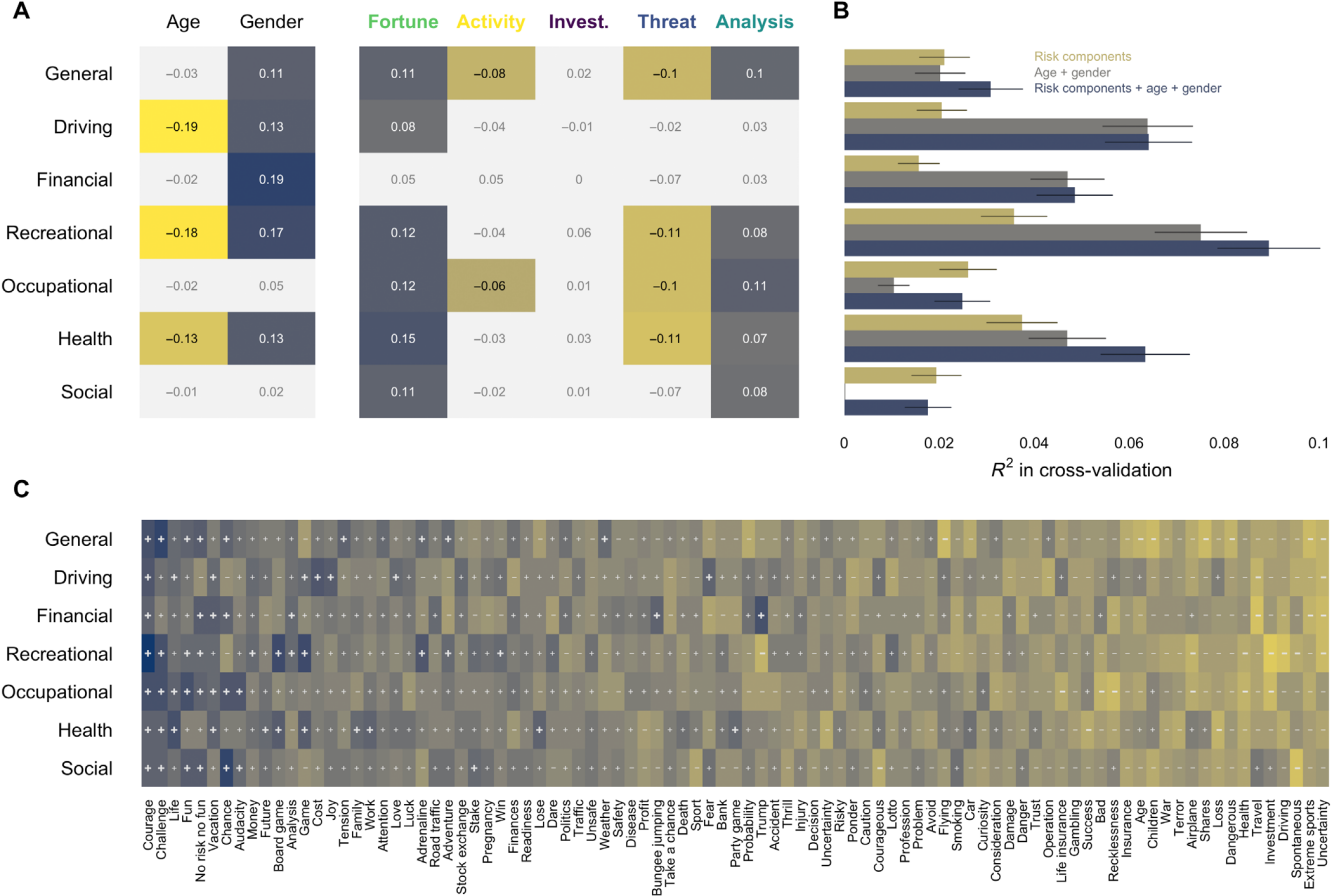
Linking the semantic representation of risk to self-reported risk taking. (**A**) Standardized coefficients for regressions predicting self-reported risk taking from a joint set of predictors including age, gender, and the similarity of people’s responses to each of the five risk components separately for each of the seven risk-taking propensity items. Cells shown in color are significant at α = 0.05. (**B**) Coefficient of determination (*R*^2^) in cross-validation achieved by models predicting self-reported risk-taking propensity either by the five risk components, by age and gender, or by all predictors combined. Errors bars reflect SEs according to the corrected resampled *t* test (*59*). (**C**) Correlation between retrieving or not retrieving a term at level 1 and the self-reported risk-taking items. Pluses and minuses reflect the direction of correlation. Bold font is used to signify correlations > ∣ 0.05∣.

To answer this question, we used linear regression to predict participants’ judgments in the seven self-reported risk-taking items, capturing risk taking in general and in the following six specific domains: driving, financial, recreational, occupational, health, and social, using age and gender as well as the similarity of people’s level 1 responses to each of the five risk components. As can be seen in Fig. 8A, this revealed significant effects for four of the five risk components. Responses related to the component Fortune and Analysis showed significant positive relationships with judgments in the general and most of the six domain-specific items, whereas responses related to the component Threat and Activity showed significant negative relationships to judgments in the general and most of the domain-specific items (see Fig. 8A). One should note that these results reveal no domain-specific patterns in the predictive validity of the components, suggesting that the latter leverage general evaluative information that is common across the domains to predict individual differences. Last, applying a cross-validation approach using elastic net regularization, we observed that a model including either both the demographic and risk component predictors or only the risk component predictors was able to reliably predict more variance relative to a model including only the demographic predictors for all risk-taking items (Fig. 8B). On the whole, these results suggest that semantic information captured by at least some of the risk components can help predict individual differences in self-reported risk taking.

We examined further the link between the semantic representation of risk and individual differences in risk taking by exploring the relation between the retrieval of individual terms and self-reported risk taking. Figure 8C shows the correlations between retrieving/not retrieving the terms and judgments in the seven risk-taking items for 91 terms that were retrieved at least 10 times (ordered by average correlation). Similar to the effects for risk components, the figure reveals that individual terms tend to be related to risk taking in a non–domain-specific way. Specifically, terms such as courage, challenge, life, or fun showed consistent positive associations to the general and all domain-specific items, whereas terms such as uncertainty, extreme sports, spontaneous, or driving showed consistent negative associations. Notably, there were very few terms that showed notable or conflicting correlations (bold ±) with individual risk-taking domains.

Last, we assessed the additional predictive power of a general word embedding model that was not specifically developed to capture the semantic representation of risk. Finding that a general word embedding model contributes further explained variance in risk taking could reveal the extent to which some aspects of individuals’ semantic representation remain untapped by the risk components identified in our work. To this end, we represented every person’s level 1 retrievals as the sum of the corresponding word vectors from the pretrained German fastText word embedding model (*40*) and then used the 300 embedding dimensions to predict their risk taking using elastic net regression. We found that the word vectors accounted, on average, for less variance than the risk components (1.9% versus 2.5%), and their inclusion in a joint model together with age, gender, and the risk components led to a small decrease in performance (4.3% versus 4.8%). These results suggest that the five risk components capture relevant aspects associated with risk-taking propensity to at least a similar extent compared to a more complex model that considers many more semantic dimensions. All in all, these results suggest that the different semantic components that we identified can be used to trace the link between individual differences in the semantic representation of risk and self-reported risk taking.

## DISCUSSION

We explored the semantic representation of risk and potential individual and group (age and gender) differences in the representation of this concept using a novel mini-snowball word association task. The task asked participants to generate words associated with the concept of risk and words associated with the initial risk associates. We then conducted network and sentiment analyses to identify a general network of risk associates and made use of this network to characterize individual differences across age and gender. In what follows, we summarize our main findings and discuss the main limitations of our work.

Our starting point was the identification of a general semantic network of risk composed of 306 primary associates of risk. This network exhibited a complex organization involving five distinct semantic components, which we labeled as Threat, Fortune, Activity, Investment, and Analysis. In line with extant theories of lay people’s representation of risk [e.g., (*7*)], the Threat component (consisting of mainly negative consequences of risk) emerged as most prominent in people’s semantic network. However, the other components accounted for more than half of people’s associations with “risk,” even when only considering first responses, which is consistent with the widely held multidimensional perspective on the psychology of risk [e.g., (*4*)]. Notably, the Fortune component seems to represent positive associations with “risk,” echoing recent calls for the need to understand positive aspects associated with risk and risk taking (*12*) as well as the intrinsically close relation between risk and reward (*41*). Nevertheless, the semantic components of our network have some correspondence to past taxonomies of risk. Specifically, the components Threat and Analysis align well with Fischhoff *et al*. (*11*) dimensions of dread and uncertainty as well as the idea that risk refers to potential loss as a consequence of an active decision (*4*). Similarly, the remaining three components—Fortune, Activity, and Investment—capture, albeit not perfectly, some of the domain-specific nature of risk taking identified in past approaches (*9*).

One unique contribution of our approach was the assessment of universal aspects of the semantic representation of risk across languages. Our results comparing the risk components identified through our novel mini-snowball approach to existing word association data for other languages, Dutch and English, suggest that the components that we identified can be traced across languages, albeit with some variation in their relative frequencies. These results suggest that there are some universal characteristics of the representation of risk, which could be a product of linguistic and ecological similarities in how humans encounter “risk” in their daily lives.

Notwithstanding the similarities concerning risk components across languages, one main goal of our approach was to evaluate potential group differences in the semantic representation of risk. Concerning age, we found a notable consistency in the prominence of the components of risk across age groups, suggesting that people of different age groups think about risk in similar terms. Yet, there were some noteworthy differences identified at the component and word level. Specifically, we found that, whereas younger adults retrieved words of the components Threat and Fortune in nearly equal proportions, there was clear divergence for older adults, with Threat being much more prominent than Fortune. These results are compatible with the idea that positive aspects of risk and risk taking change across the life span (*10*, *12*, *17*). Furthermore, when we inspected age-related changes at the word level, we observed a number of differences, with words concerning mobility and health being prominent among older adults and words concerning entertainment and financial prospects being prominent among younger adults. Thus, despite strong macroscopic-level similarities in the semantic representation of risk, there exist systematic differences across age that could be linked to ecological and life circumstances of different age groups (*10*, *16*).

Concerning gender, we again observed consistency in the prominence of the different risk components, suggesting that both males and females think about risk in similar terms. Nevertheless, differences emerged for Threat and Fortune, with the former being more prominent among females and the latter more prominent among males. Thus, similar to older adults, females seem to consider the negative consequences of risk more and the positive ones less frequently than males. Furthermore, when we inspected gender-related changes at the word level, we observed a number of differences, for example, with “pregnancy” being considerably more prominent among female respondents. Thus, despite a similar level of similarities in the semantic representation of risk, there is also evidence of some systematic differences across gender.

Last, we estimated the potential link between people’s risk representations and self-reported risk-taking propensity in general and in specific domains (e.g., recreational, health, driving, and financial). We observed that a model using the five risk components significantly predicted individual differences in self-reported risk taking over and beyond the age and gender of a person. The links were not domain specific and unequally distributed across risk components, such that higher proportions of responses relating to, in particular, Fortune and Analysis, were generally associated with higher risk-taking propensities, whereas responses relating to Threat were associated with lower risk-taking propensities. Overall, these results suggest that information about the semantic representation of risk can be leveraged to predict individual differences over and beyond what can be done with basic demographic information. More generally, these links, although correlative in nature, are consistent with the idea that associative retrieval processes underlie self-reported risk judgments and therefore can be partly determined by individual differences in the semantic representation or retrieval of risk information [e.g., (*16*)].

### Limitations and future directions

Our work has a number of limitations that merit discussion. First, our task uses a lexical approach based on associations of only five associates per individual (with the remaining responses being used to characterize the links between risk associates). Although the similarity of findings across our study and SWOW data suggests some robustness across elicitation methods, the small set of responses per person likely limits the richness of the representation and may neglect lower-frequency associates that could, in principle, be retrieved. Furthermore, it is possible that other approaches, including more focused elicitation methods using experimenter-generated stimuli or dimensions, could provide more fine-grained associations and therefore richer representations of risk. Future work should compare different forms of elicitation to assess the generalizability of our findings in terms of the range of components extracted and other findings concerning the semantic representation of risk.

Second, our elicitation method cannot provide truly individual semantic representations. For this purpose, richer sets of data would be needed, requiring more intensive designs [e.g., (*42*–*44*)]. Such approaches would be instrumental to improving predictions at the individual level, thus fulfilling the promise of uncovering the role of semantic representations for individual differences in risk-related constructs and other domains. Recent work has suggested that lexical network approaches may be powerful tools to make predictions about population-level risk perception of novel risks (*45*), and it would be interesting to assess the possibility of extending our approach to make similar but personalized predictions for single individuals across all areas of knowledge [cf. (*43*)].

Third, our identification of group differences, such as those related to age or gender, does not clarify the origins of these differences. For example, our data suggest age differences in the types of activities spontaneously associated with risk, such as recreational risk taking. Several aging theories could be used to explain such a change, including those that make assumptions about motivational changes, concerning the role of activation and excitement (*46*) or loss avoidance (*47*). To distinguish such hypotheses, other survey methods that directly capture the motivation and goals associated with certain concepts could be a better or complementary approach (*10*).

Fourth, our work does not investigate the mechanisms linking the empirical associations and self-reported risk taking. One possibility is that the predictive power of empirical associations is driven by long-term group differences in semantic representations. Recent work has identified systematic differences in the semantic networks of groups and individuals (*48*) and suggested that they can contribute to individual differences in behavior (*49*, *50*). Alternatively, or in addition, situational activation of mental representations can also contribute to these individual and group differences. Additional work that examines ecological and situation-specific risk taking could prove helpful in teasing apart these contributions.

Fifth and last, we only assessed the link between semantic representations and a single elicitation method for risk preference (i.e., self-reported propensity). Future work could investigate additional operationalizations of risk preference including behavioral measures of risk preference. We believe that there is likely additional work that is needed to develop suitable measures before such a study can be conducted. Currently, most behavioral measures of risk preference show somewhat poor psychometric characteristics, such as low reliability or convergent validity (*51*, *52*), and do not show systematic associations to demographic characteristics like age and gender (*17*). To the extent that future work can provide reliable estimates of both individual semantic representations of risk and operationalizations of risk preference, it would be interesting to assess whether individual differences in semantic representation of risk can account for the limited convergent validity of different operationalizations of risk preference [e.g., (*51*, *53*)] by assessing whether the latter are interpreted differently by different individuals. Our mini-snowball procedure could potentially be adapted to investigate the perceptions associated with such behavioral measures by probing words associated with such measures.

Despite these limitations, our results highlight the promise of a controlled free association approach, in particular, our mini-snowball paradigm, to shed light on features of psychology that are less accessible by text- or survey-based approaches. We are convinced that this and similar approaches could be used to further illuminate people’s mental representations of risk and other psychological or social constructs. Our approach could be particularly useful for those constructs for which considerable heterogeneity of perspectives or even polarization exist, from vaccination to sustainability.

To conclude, our work contributes to a better understanding of the semantic representation of risk. Our results are in line with past findings, suggesting that risk is a multifaceted construct with a rich set of associates that pervades several areas of life. Our work extends our knowledge of the semantic representation of risk by emphasizing the coexistence of both negatively and positively valenced components, questioning the currently predominant focus on negative aspects of risk. Our results also highlight the importance of understanding individual and group differences such as age and gender in the semantic representation of risk and showcase the promise of using controlled free association methodology as a means to characterize lay people’s representation of complex psychological constructs. One main implication of our work is that the measurement of risk-related constructs, such as risk perception or risk taking, requires an understanding of the many meanings of “risk” and how they differ between individuals and groups.

## MATERIALS AND METHODS

### Participants

A total of 1205 (602 female, 50%) participants completed the online study in November 2016. Participants were between 18 and 87 years of age (*M* = 47.7 and SD = 16.6). Participants were part of a panel and recruited by a survey company for market research purposes (www.forsa.de). Participants were sampled evenly from six age bins (18 to 27, 28 to 37, 38 to 47, 48 to 57, 58 to 67, and 68+), designed to be representative of the German population. The study took an average of 12 min (median = 10 min), and participants received a 2-EUR Amazon voucher for their participation.

### Measures

#### Word association task

Word associations were collected using a mini-snowball word association task. Participants first gave five associations to the word risk and then, on separate pages, five associations to each of the five associates of the word risk in the order that they were initially produced for a total of 30 associates per participant. Participants were instructed to name the first five words that came to mind when thinking exclusively about the respective cue. They were further instructed to answer as spontaneously as possible to avoid repeating associations while responding to the same cue and to avoid responding in full sentences. The elicitation of multiple associates per cue and the instructions are inspired by the protocol of the SWOW projects (*24*, *44*). These also recruit a multiresponse format based on findings showing that this substantially increases the breadth and heterogeneity of responses (*24*, *54*), which is important for both obtaining a wide coverage of the semantic space and, in our case, the study of individual and group differences.

Participants’ responses were subjected to manual spelling correction before analysis. Overall, 2.78% of all responses were corrected with a median string distance between the original and corrected word of 1 (restricted Damerau-Levenshtein distance) [e.g., (*55*)]. An additional 0.13% of responses either were identified as nonwords, e.g., “djjebdb,” or clearly expressed failure to produce an associate, e.g., “no idea” (German: “keine Ahnung”) or “?”. These responses were removed from all further analyses.

#### Inferring sentiment

Sentiment was determined on the basis of the SentiWS dictionary (*38*). Of the 307 words in the general network of risk, only 115 were contained in the SentiWS dictionary. To be able to analyze sentiment for a maximal number of words, the average sentiment of level 2 responses was used to infer the sentiment of unavailable level 1 sentiment. Specifically, sentiment was defined as the average between the word’s sentiment and the average sentiment of its associates or only the latter, whenever the sentiment of the wave 1 word itself was unavailable. For the 115 words for which sentiment values were directly available, we found a correlation of *r* = 0.56 between the word’s sentiment and its inferred sentiment score based on the sentiment of its associates, suggesting that the average sentiment of associates provides a reliable indicator of the sentiment of a target word. This approach delivered sentiment scores for 304 of the 307 words.

#### Risk-taking propensity

Risk-taking propensity was measured using the seven self-report risk items from the German Socio-Economic Panel (SOEP) (*56*). The first item asks participants to judge “Are you in general a risk-seeking person or do you seek to avoid risks?” (translated from German) on a scale from 0 (not risk seeking at all) to 10 (very risk seeking). The following six items asked the same question and provided the same scale, but replaced “in general” with text referring to one of six different risk-taking domains including driving, financial investments, leisure and sports, professional career, health, and trusting strangers.

#### Procedure

Participants were contacted by a survey company to participate in the computerized online study. First, participants were informed about the goals and specifics of the study and gave informed consent. Then, participants completed the word association task followed by the SOEP risk-taking propensity task. The study was approved by the Institutional Review Board of the Faculty of Psychology at the University of Basel.

### Multilingual analysis

Free association data were obtained from the publicly available data from the English and Dutch SWOW projects (see https://smallworldofwords.org/de/project/research). Both datasets include 300 associates for each of several thousand different cues, including 244 (Dutch) and 253 (English) terms, respectively, which could be matched to the 307 terms in our network of risk. To match terms between languages, we relied on the German-English translations presented in the Supplementary Materials and followed an analog translation procedure for German-Dutch.

## Supplementary Materials

### This PDF file includes:

Sections S1 to S5 Tables S1 and S2 Figs. S1 to S4 References
