# Supplementary material for: On the semantic representation of risk
Source: Sci Adv. 2022 Jul 8;8(27):eabm1883. doi: 10.1126/sciadv.abm1883 (PMC9269897; doi:10.1126/sciadv.abm1883)
Supplement: Supplementary file 1 — Sections S1 to S5 Tables S1 and S2 Figs. S1 to S4 References [file sciadv.abm1883_sm.pdf]

Science Advances
AAAS

Supplementary Materials for

On the semantic representation of risk

Dirk U. Wulff *et al.*

Corresponding author: Dirk U. Wulff, dirk.wulff@gmail.com

Sci. Adv. **8**, eabm1883 (2022)
DOI: 10.1126/sciadv.abm1883

This PDF file includes:

Sections S1 to S5
Tables S1 and S2
Figs. S1 to S4
References

S1: Word translation

The translation of German Level 1 responses to English was carried out in a two-stage procedure. First, words were translated automatically (as a newline-separated list) using the free DeepL translation service. Then, in a second step, the accuracy of translations was confirmed manually. This was necessary especially to appropriately interpret polysemious words such as "einsatz", which, among others, can mean "mission" (suggested by DeepL) or "stake" (used after manual correction). See Table S1 and Table S1 for the full list of translations.

Table S1

German to English translation

German	English	German	English	German	English	German	English
risiko	risk	adrenalin	adrenaline	skifahren	skiing	geschäft	business
angst	fear	wette	bet	freudig	joyful	dunkelheit	darkness
arbeit	work	börse	stock exchange	möglichkeit	opportunity	zweifel	doubt
freude	joy	spekulation	speculation	abwägen	ponder	ansteckung	infection
mut	courage	arbeitsplatz	workplace	kalkulierbar	calculable	stress	stress
gefahr	danger	leichtsinn	recklessness	kosten	cost	überlegung	consideration
rauchen	smoking	unsicher	unsafe	bergsteigen	mountaineering	geldverlust	money loss
tod	death	wasser	water	minimieren	minimize	liebe	love
gesundheit	health	wetter	weather	gesellschaftsspiel	party game	umwelt	environment
unfall	accident	herbst	autumn	wim thoelke	wim thoelke	spontaneität	spontaneity
ungewiss	uncertain	sicherheit	safety	absicherung	hedging	versicherungen	insurance
spiel	game	straßenverkehr	road traffic	haus	house	flucht	escape
altersarmut	age poverty	lotto	lotto	absturz	crash	niedrig	low
schaden	damage	fallschirmsprung	skydiving	banken	banks	freundschaft	friendship
verlust	loss	analyse	analysis	schwer	heavy	klima	climate
schlecht	bad	rente	pension	beruf	profession	kind	child
versicherung	insurance	wagemut	audacity	verantwortung	responsibility	scheitern	failure
vorsicht	caution	chance	chance	risikobereitschaft	risk appetite	aufpassen	pay attention
zwo, eins, risiko.	two, one, risk.	zufall	coincidence	faktor	factor	unbekanntes	unknown
wagnis	dare	fußball	soccer	schnee	snow	ängstlich	fearful
verkehr	traffic	ehe	marriage	schmerzen	pain	veränderung	change
gefahren	dangers	riskant	risky	negativ	negative	nutzen	benefit
darkwin duck	darkwin duck	autofahren	driving	bedenken	concern	beziehung	relationship
brettspiel	board game	fliegen	flying	unbekannt	unknown	möglichkeiten	opportunities
bewertung	rating	schutz	protection	abschätzen	estimate	lust	desire
leben	life	motorradfahren	motorcycling	aktie	share	notwendig	necessary
eingehen	take a chance	spontan	spontaneous	job	job	ablehnen	reject
fun	fun	aufregung	excitement	projekt	project	abschätzung	estimate
einschätzung	assessment	klettern	climbing	alkohol	alcohol	investition	investment
geld	money	mutig	courageous	unvermeidbar	unavoidable	autobahn	highway
gefährlich	dangerous	glatteis	black ice	unkalkulierbar	incalculable	geschwindigkeit	speed
extremsport	extreme sports	ungewissheit	uncertainty	zeit	time	unwissenheit	ignorance
sport	sport	glück	luck	prüfen	check	risikominimierung	risk minimization
fahren	driving	hilfe	help	weihnachten	christmas	reich	rich
einschätzen	estimate	politik	politics	wagen	trolley	vielleicht	perhaps
unsicherheit	uncertainty	operation	operation	vermeidbar	avoidable	wirtschaft	economy
auto	car	krieg	war	unberechenbar	unpredictable	no risk no fun	no risk no fun
bereitschaft	readiness	bank	bank	etwas wagen	dare something	hoffnung	hope
poker	poker	herausforderung	challenge	spannend	exciting	wertpapiere	securities
glücksspiel	gambling	terror	terror	verletzung	injury	verbrechen	crime
krankenhaus	hospital	gefährdung	threat	autorennen	car race	freizeit	free time
neugier	curiosity	urlaub	vacation	nervenkitzel	thrill	finanziell	financial
management	management	achtung	attention	warnung	warning	das leben	life
lebensversicherung	life insurance	geldanlagen	investments	reisen	travel	teuer	expensive
armut	poverty	sonne	sun	not	emergency	jugend	youth
gewagt	daring	sturz	fall	terrorismus	terrorism	spielen	play
schule	school	schwangerschaft	pregnancy	chancen	opportunities	strasse	street
aktien	shares	vermeiden	avoid	kinder	children	vertrauen	trust
segeln	sailing	fonds	funds	erfahrung	experience	überschätzung	overestimation
risikoanalyse	risk analysis	vermeidung	avoidance	wahrscheinlichkeit	probability	höhe	altitude
unbehagen	discomfort	problem	problem	versuch	attempt	neugierde	curiosity
spaß	fun	schmerz	pain	reiz	stimulus	straße	road
gewinn	profit	schnelligkeit	speed	fehler	error	partnerschaft	partnership
krankheit	disease	bedrohung	threat	abenteuerurlaub	adventure vacation	nachteil	disadvantage
hoch	high	fallschirmspringen	skydiving	probleme	problems	schwierig	difficult
das spiel	the game	alter	age	gewinnen	win	entscheidungen	decisions
groß	great	rendite	return	verlieren	lose	überwindung	overcoming
spannung	tension	zocken	gamble	schicksal	fate	blumen	flowers
bungee-jumping	bungee jumping	flugzeug	airplane	entscheidung	decision	lügen	lying
trump	trump	einsatz	stake	rot	red	unternehmen	company

Table S2

German to English translation (continued)

German	English	German	English	German	English	German	English
krebs	cancer	berufswechsel	change of job	überlegen	reflect	waghalsig	daring
gruppe	group	jobwechsel	job change	pech	bad luck	existenz	existence
geldanlage	investment	islam	islam	zukunft	future	unwohlsein	discomfort
abwägung	consideration	weihnachtsmarkt	christmas market	unnötig	unnecessary	anspannung	tension
frieden	peace	neugierig	curious	verluste	losses	fahrrad	bicycle
finanzen	finances	freeclimbing	freeclimbing	übermut	overconfidence	roulette	roulette
bereit	ready	gut	good	kasino	casino	einbruch	burglary
abenteuer	adventure	schnell	fast	unwetter	storm	aufmerksamkeit	attention
kapital	capital	sex	sex	neues	new	abwarten	wait
nachdenken	contemplate	nein	no	leiter	ladder	betrug	fraud
motorrad	motorcycle	familie	family	aufregend	exciting	lohnend	rewarding
kristall	crystal	wetten	betting	sorge	worry	hobby	hobby
arbeitslosigkeit	unemployment	erfolg	success	bunt	colorful	ängste	fears
feuer	fire	riskieren	risk	sucht	addiction	ungeduld	impatience
herzinfarkt	heart attack	casino	casino	ärger	trouble	fünfzig fünfzig	fifty fifty

S2: Network stability analysis

To assess the distinctiveness of our network components, we utilized a cluster stability approach based on bootstrap sampling. Specifically, for each individual Level 1 word in the network, we repeatedly took 1,000 samples with replacement from the word's Level 2 responses. For each set of samples, we then created a network and extracted components using the process depicted in Figure S1. For a component that is both distinct and stable, we expect that words belonging to the component would be more likely to share components with other words from the original component than with words from another component across the bootstrap samples.

Figure S1 shows each word's (each row) probability of sharing a component with words from its own and the other four components in a way that accounts for cluster sizes. We found that 208 of 307 words, which are shown in the original component color, were more likely to share a component with words of the same original component (than with words from other components). This measure of consistency was high for the component *Threat* (50 out of 57 words), *Investment* (50/64), and *Fortune* (72/82). By comparison, the components *Activity* (30/64) and, especially, *Analysis* (6/40) showed lower consistency. Among the words in these two clusters that did not preferably share a cluster with its own words, many preferably shared clusters with words of Threat. This was the case for 17 out of 34 words in *Activity* and 28 out of 35 words in Analysis. This suggests a certain level of semantic overlap, especially for *Threat* and *Activity*.

Overall, it should be noted that all words were found to frequently share clusters with words from other clusters. This highlights the possibility of alternative clusterings of our data that we address in the following section. That said, it seems probable that the words belonging to components *Threat*, *Investment*, and *Fortune* would likely also share memberships in alternative clusterings. Moreover, the most representative words for the components, e.g., "danger" for *Threat* or "happiness" for *Fortune*, showed particularly high levels of stability, suggesting that the corresponding components possess a robust thematic core. For *Analysis* and *Activity* such cores are absent in our clustering.

Finally, unsupervised techniques such as modularity detection should not be viewed as discovering an underlying *true* structure, but rather as delivering useful and, ideally, robust summarization that facilitates an intuitive understanding of the data [*37*]. We believe that our clustering achieves this and can, as we describe below, be seen as a robust way of summarizing the data.

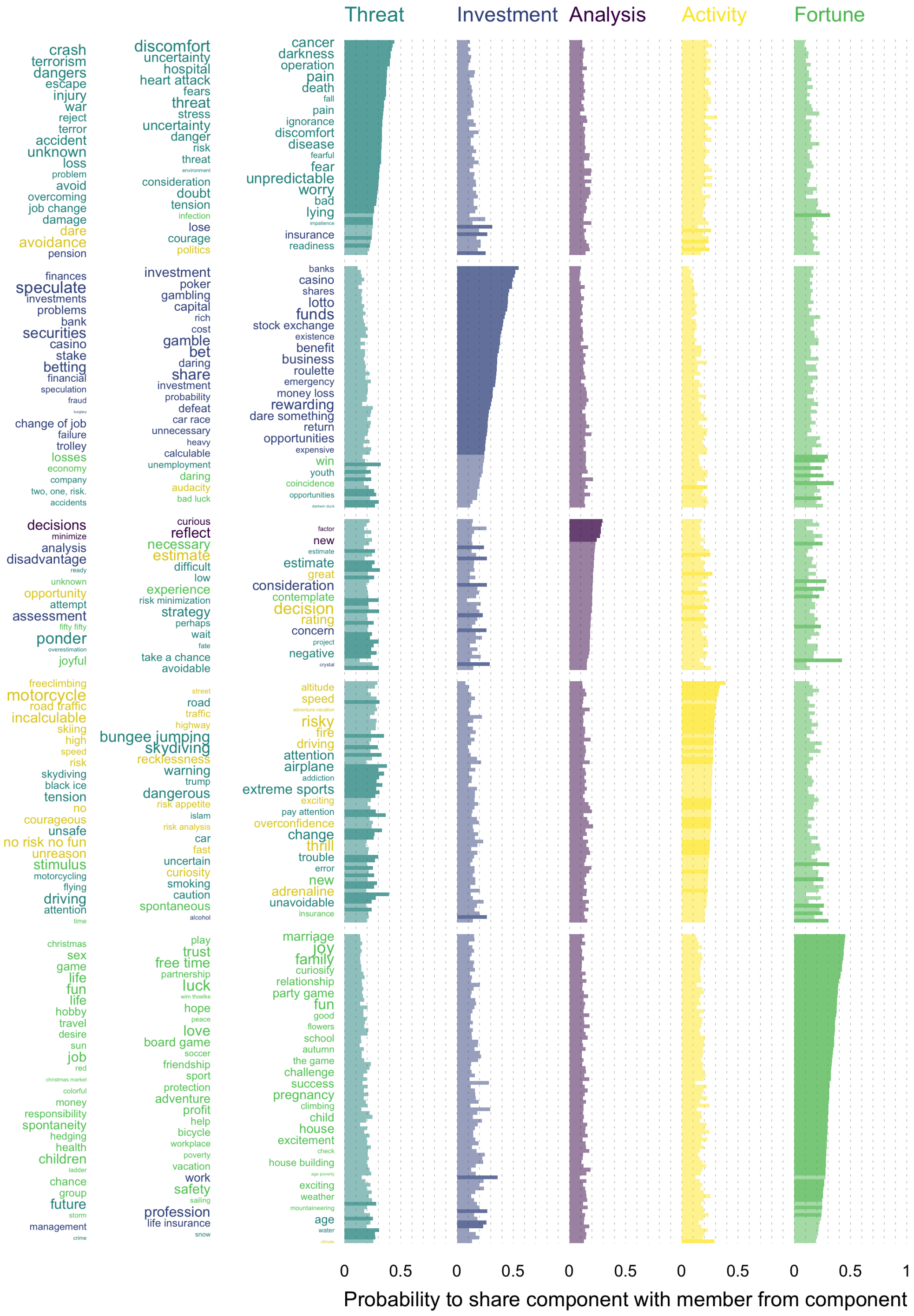

Figure S1

Network stability. Each row shows the probability of a word sharing clusters with words from each of the five clusters across 1,000 bootstrap samples. Probabilities are shown as vertical bars for each of the five clusters (columns). Opaque vertical bars indicate that the probability is highest for the original cluster. Word labels are colored according to its preferred cluster, i.e., the cluster whose words most frequently share cluster membership with the word. Label size varies as a function of the word's importance in each of the clusters as determined by PageRank.

S3: Alternative clustering solutions

To shed light on the robustness of our results with respect to clustering algorithms and the number of clusters extracted, we used an alternative approach to assess the similarities of Level 1 responses using hierarchical clustering, which in contrast to the Louvain approach reported in the main text, leaves the choice of number of clusters to the analyst. Specifically, we used the agglomerative variant and Ward's minimum variance linkage method to derive clustering solutions for two up to ten different clusters. Figure S2 shows the agglomerative progression from ten to two clusters with each cluster shown as a word cloud.

The hierarchical clustering algorithm produced clusters at different levels that clearly match the components identified by the Louvain algorithm. The components *Threat*, *Fortune*, and *Investment* emerge consistently at the left-most, second to right-most, and right-most arms of the Figure, while the component *Analysis* is included in the central-most arm and emerges as a separate cluster at the bottom. The only component that does seem to be captured by distinct clusters is *Activity*, which is in line with the stability results presented above and suggests that the *Activity* consists of mixed clusters capturing various aspects of *Threat*. The hierarchical clustering furthermore reveals several intriguing hierarchical relationships between the clusters. First, the component *Investment* appears to consist of two sub-components capturing the distinction between investing and betting that also has been documented in previous work on risk taking [9]. Second, the component *Fortune* appears to consist of two sub-components capturing fun and games on the one hand, and safety and insurance on the other hand. Third, the component *Threat* appears to consist of several sub-components including distinct sub-components focusing on transportation-related, job-related, or political threats.

It is important to note that the hierarchical clustering approach relies on a similar yet different objective function from the Louvain algorithm employed in the main analysis, implying that the two methods cannot be expected to always produce identical results. The fact that the hierarchical clustering approach produces similar clusters (at a specific hierarchical level) to those identified by the Louvain algorithm suggests that the five clusters we consider in the main paper can be identified robustly across different clustering approaches.

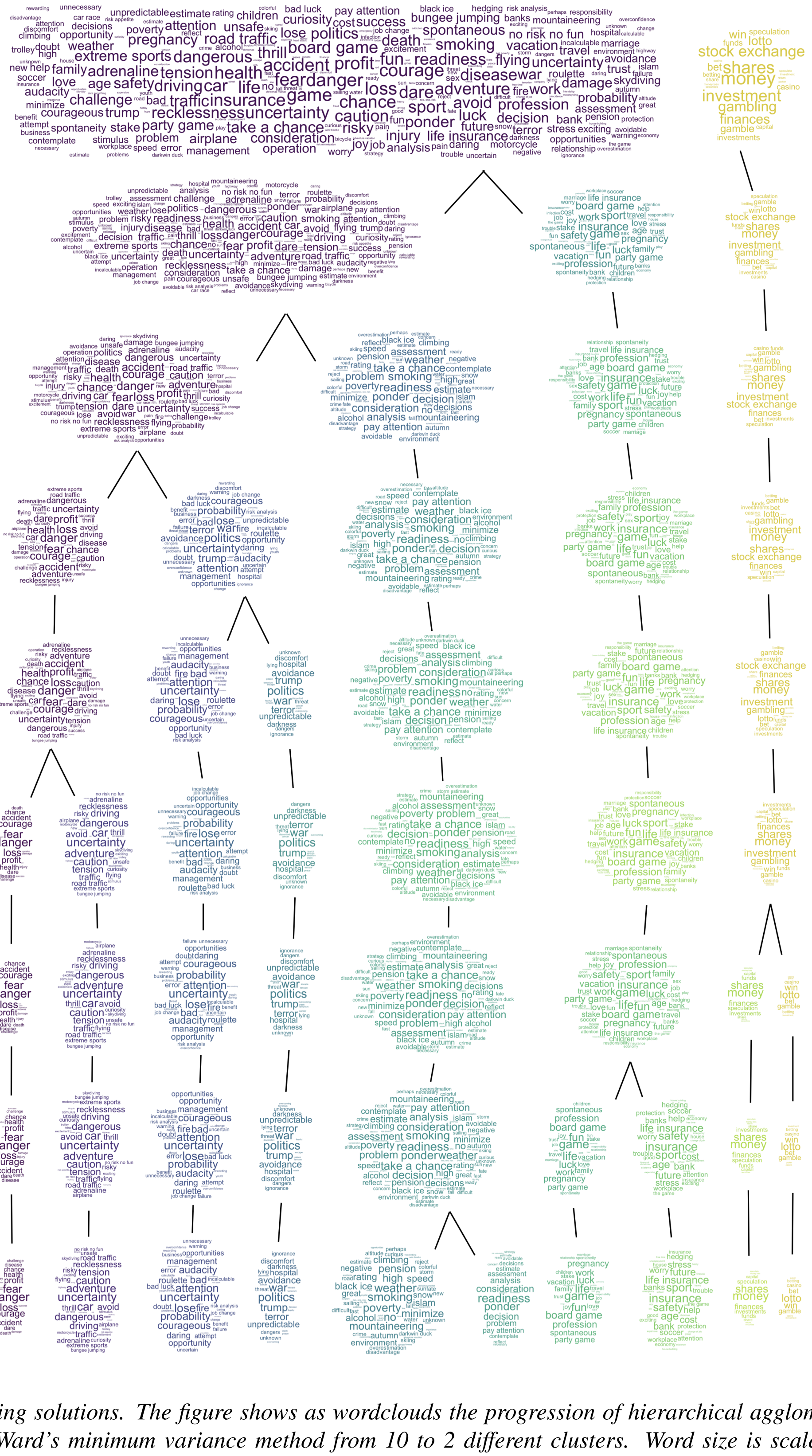

Figure S2

Hierarchical clustering solutions. The figure shows as wordclouds the progression of hierarchical agglomerative clustering solutions based on Ward's minimum variance method from 10 to 2 different clusters. Word size is scaled by the retrieval frequency rank within each cluster.

S4: Sentiment and Risk Similarity

To further explore potential commonalities and differences between the semantic network of risk between languages, we also characterized the five components of risk in the Dutch and English networks with respect to sentiment and risk similarity following the same approach as in the main analysis for German presented in the article. Sentiment was determined using language specific valence dictionaries in Dutch [*60*] and English [*61*]. Risk similarity was determined using the Jacccard similarity between the word risk and all other words in the respective language. The results show that the patterns of component sentiment and risk similarities in the two languages closely resemble those presented for German (see Figure 4D). The components *Threat* and *Fortune* again occupy the extreme position on the sentiment spectrum with *Threat* having the most negative and *Fortune* having the most positive sentiment. Note that here sentiment is measured on a different scale with mid points of 4 (Dutch) and 5 (English) rather than 0 as in the main analysis, due to differences in response formats in the generation of the different sentiment dictionaries. Furthermore, the component *Threat* is again high in risk similarity, clearly exceeding the risk similarity of the component *Fortune*, the component *Analysis*, and, in the case of English, the component *Activity*. However, in contrast to the results obtained for German, the component *Investment* was found to have the strongest risk similarity in both Dutch and English.

All in all, these results further underpin the commonalities in the semantic representation of risk in German, Dutch, and English, while also revealing small, but notable differences in the relative placement of specific components (e.g., *Investment*) that could reflect intercultural differences between languages.

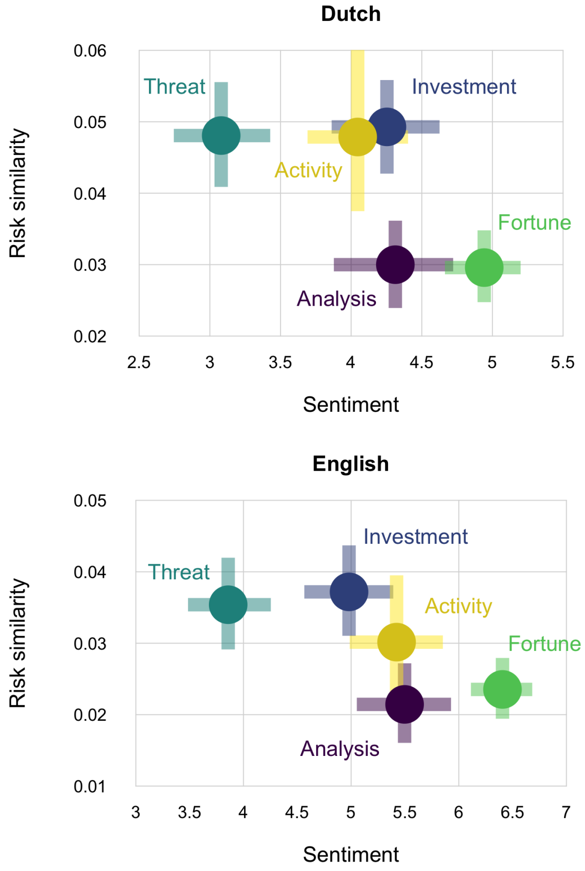

Figure S3

The figures shows the average sentiment and proximity to risk for each of the five components in Dutch and English. Error bars indicate bootstrapped 95% confidence intervals.

S5: Demographic differences in risk associations

To further investigate demographic differences in the semantic representation of risk, we analyzed people's free associations to risk as a function of education level, employment status, and relationship status in addition to age and gender. Figure S4 shows the retrieval proportion of retrievals from each of the five risk components, as well as the retrieval proportions for each Level 1 associate of risk that was retrieved at least 10 times (n = 91), separately for each demographic group. The analysis revealed considerable demographic differences in the relative frequency of risk components for employment status (sd = .025) and marital status (sd = .020), but not education level (sd = .011), as indicated by the average standard deviation of groups across risk components and compared to those of age (sd = .19) and gender (sd = .024). Furthermore, group differences in employment and marital status appeared to be similar to those observed for age. Specifically, similar to older participants, married and pensioned participants tended to show higher levels of *Threat*, lower levels of *Fortune*, and higher levels of *Activity*, as compared to the respectively other groups. These similarities can at least partially be explained by high correlations between those two demographic variables and age, potentially suggesting that the age-related differences we identify may be the product of individuals' social roles rather than age per se. Note that our study design only sampled age and gender in balanced fashion, thus limiting variation in group differences for other demographic variables.

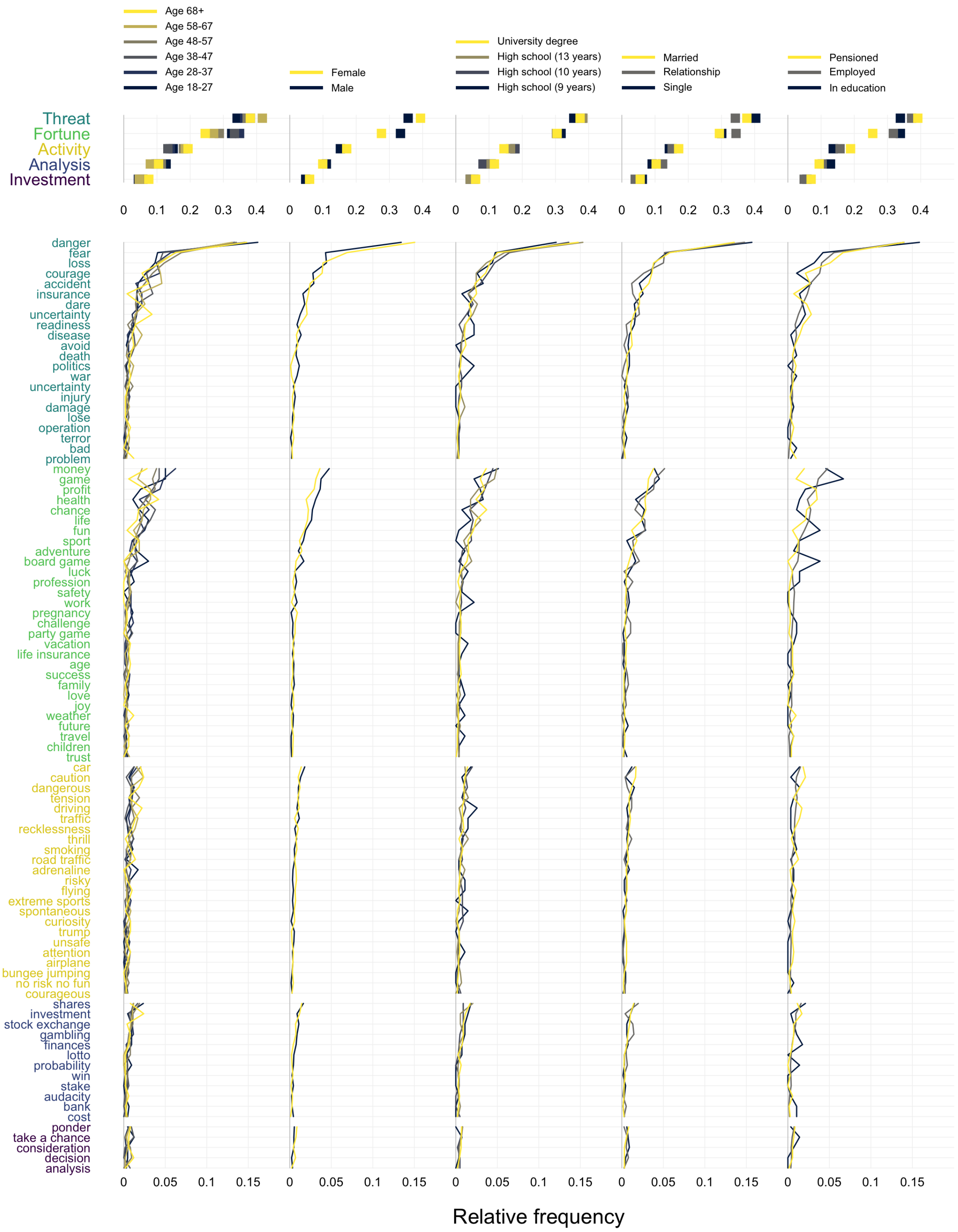

Figure S4

Demographic differences in risk associations. Relative retrieval frequencies as a function of demographic age (first column), gender (second column), education (third column), marital status (fourth column), and employment status (fifth column).

REFERENCES AND NOTES

1. C. Starr, Social benefit versus technological risk: What is our society willing to pay for safety? *Science* **165**, 1232–1238 (1969).

2. Y. Li, T. Hills, R. Hertwig, A brief history of risk. *Cognition* **203**, 104344 (2020).

3. T. Aven, The risk concept—Historical and recent development trends. *Reliab. Eng. Syst.Saf.* **99**, 33–44 (2012).

4. M. Boholm, The semantic field of risk. *Saf. Sci.* **92**, 205–216 (2017).

5. S. Boudia, N. Jas, Introduction: Risk and 'risk society' in historical perspective. *Hist. Technol.* **23**, 317–331 (2007).

6. T. Schonberg, C. R. Fox, R. A. Poldrack, Mind the gap: Bridging economic and naturalistic risk-taking with cognitive neuroscience. *Trends Cogn. Sci.* **15**, 11–19 (2011).

7. P. Slovic, Perception of risk. *Science* **236**, 280–285 (1987).

8. T. Dohmen, A. Falk, D. Huffman, U. Sunde, J. Schupp, G. G. Wagner, Individual risk attitudes: Measurement, determinants, and behavioral consequences. *J. Eur. Econ. Assoc.* **9**, 522–550 (2011).

9. E. U. Weber, A.-R. Blais, N. E. Betz, A domain-specific risk-attitude scale: Measuring risk perceptions and risk behaviors. *J. Behav. Decis. Mak.* **15**, 263–290 (2002).

10. R. D. Ravert, L. M. Murphy, M. B. Donnellan, Valuing risk: Endorsed risk activities and motives across adulthood. *J. Adult Dev.* **26**, 11–21 (2019).

11. B. Fischhoff, P. Slovic, S. Lichtenstein, S. Read, B. Combs, How safe is safe enough? A psychometric study of attitudes towards technological risks and benefits. *Policy Sci.* **9**, 127–152 (1978).

12. N. Duell, L. Steinberg, Positive risk taking in adolescence. *Child Dev. Perspect.* **13**, 48–52 (2019).

13. E. M. Bonem, P. C. Ellsworth, R. Gonzalez, Age differences in risk: Perceptions, intentions and domains. *J. Behav. Decis. Mak.*, **28**, 317–330 (2015).

14. C. Marris, I. Langford, T. Saunderson, T. O'Riordan, Exploring the "psychometric paradigm": Comparisons between aggregate and individual analyses. *Risk Anal.*, **17**, 303–312 (1997).

15. M. Siegrist, C. Keller, H. A. Kiers, A new look at the psychometric paradigm of perception of hazards. *Risk Anal.* **25**, 211–222 (2005).

16. R. C. Arslan, M. Brümmer, T. Dohmen, J. Drewelies, R. Hertwig, G. G. Wagner, How people know their risk preference. *Sci. Rep.* **10**, 15365 (2020).

17. R. Frey, D. Richter, J. Schupp, R. Hertwig, R. Mata, Identifying robust correlates of risk preference: A systematic approach using specification curve analysis. *J. Pers. Soc. Psychol.* **120**, 538–557 (2021).

18. J. J. Rolison, Y. Hanoch, S. Wood, P.-J. Liu, Risk-taking differences across the adult life span: A question of age and domain. *J. Gerontol.B Psychol. Sci. Soc. Sci.*, **69**, 870–880 (2014).

19. S. De Deyne, D. J. Navarro, A. Perfors, G. Storms, Structure at every scale: A semantic network account of the similarities between unrelated concepts. *J. Exp. Psychol. Gen.* **145**, 1228–1254 (2016).

20. S. Mollin, Combining corpus linguistic and psychological data on word co-occurrences: Corpus collocates versus word associations. *Corpus Linguist. Linguist. Theory* **5**, 175–200 (2009).

21. H. Vankrunkelsven, S. Verheyen, G. Storms, S. De Deyne, Predicting lexical norms: A comparison between a word association model and text-based word co-occurrence models. *J. Cogn.* **1**, 45 (2018).

22. S. De Deyne, A. Perfors, D. J. Navarro, Predicting human similarity judgments with distributional models: The value of word associations, in *Proceedings of the COLING 2016, the 26th International Conference on Computational Linguistics: Technical papers*, (COLING, 2016), pp. 1861–1870.

23. T. Mikolov, E. Grave, P. Bojanowski, C. Puhrsch, A. Joulin, Advances in pre-training distributed word representations. arXiv:1712.09405 [cs.CL] (26 December 2017).

24. S. De Deyne, D. J. Navarro, A. Perfors, M. Brysbaert, G. Storms, The "small world of words" English word association norms for over 12,000 cue words. *Behav. Res. Methods* **51**, 987–1006 (2019).

25. C. S. Q. Siew, D. U. Wulff, N. M. Beckage, Y. N. Kenett, Cognitive network science: A review of research on cognition through the lens of network representations, processes, and dynamics. *Complexity* **2019**, 2108423 (2019).

26. Y. N. Kenett, N. M. Beckage, C. S. Q. Siew, D. U. Wulff, Cognitive network science: A new frontier. *Complexity* **2020**, 6870278 (2020).

27. S. Ioffe, Improved consistent sampling, weighted Minhash and L1 sketching, in *Proceedings of the 2010 IEEE International Conference on Data Mining* (IEEE, 2010), pp. 246–255.

28. A. Strehl, J. Ghosh, R. Mooney, Impact of similarity measures on web-page clustering, in *Workshop on Artificial Intelligence for Web Search* (AAAI Technical Report WS-00-01, AAAI, 2000), pp. 58–64.

29. A. Huang, Similarity measures for text document clustering, in *Proceedings of the Sixth New Zealand Computer Science Research Student Conference (NZCSRSC2008),* (NZCSRS, 2008), pp. 9–56.

30. S. M. Saad, S. S. Kamarudin, Comparative analysis of similarity measures for sentence level semantic measurement of text, in *Proceedings of the 2013 IEEE International Conference on Control System, Computing and Engineering* (IEEE, 2013), pp. 90–94.

31. V. D. Blondel, J.-L. Guillaume, R. Lambiotte, E. Lefebvre, Fast unfolding of communities in large networks. *J. Stat. Mech.* **2008**, P10008 (2008).

32. P. Miasnikof, A. Y. Shestopaloff, A. J. Bonner, Y. Lawryshyn, P. M. Pardalos, A density based statistical analysis of graph clustering algorithm performance. *J. Complex Netw.* **8**, cnaa012 (2020).

33. N. Williams, G. Arnulfo, S. H. Wang, L. Nobili, S. Palva, J. M. Palva, Comparison of methods to identify modules in noisy or incomplete brain networks. *Brain Connect.*, **9**, 128–143 (2019).

34. C. Pradana, S. Kusumawardani, A. Permanasari, Comparison clustering performance based on Moodle log mining. *IOP Conf. Ser. Mater. Sci. Eng.*, **722**, 012012 (2020).

35. S. Emmons, S. Kobourov, M. Gallant, K. Börner, Analysis of network clustering algorithms and cluster quality metrics at scale. *PLOS ONE* **11**, e0159161 (2016).

36. C. Hennig, What are the true clusters? *Pattern Recognit. Lett.* **64**, 53–62 (2015).

37. J. M. Haslbeck, D. U. Wulff, Estimating the number of clusters via a corrected clustering instability. *Comput. Stat.* **35**, 1879–1894 (2020).

38. R. Remus, U. Quasthoff, G. Heyer, Sentiws-a publicly available German-language resource for sentiment analysis, in *Proceedings of the Seventh International Conference on Language Resources and Evaluation (LREC'10)*, (European Language Resources Association, 2010).

39. S. De Deyne, G. Storms, Word associations: Norms for 1,424 Dutch words in a continuous task. *Behav. Res. Methods*, **40**, 198–205 (2008).

40. E. Grave, P. Bojanowski, P. Gupta, A. Joulin, T. Mikolov, Learning word vectors for 157 languages. arXiv:1802.06893 [cs.CL] (19 February 2018).

41. T. J. Pleskac, R. Hertwig, Ecologically rational choice and the structure of the environment. *J. Exp. Psychol. Gen.* **143**, 2000–2019 (2014).

42. A. S. Morais, H. Olsson, L. J. Schooler, Mapping the structure of semantic memory. *Cogn. Sci.* **37**, 125–145 (2013).

43. D. U. Wulff, S. De Deyne, S. Aeschbach, R. Mata, Using network science to understand the aging lexicon: Linking individuals' experience, semantic networks, and cognitive performance. *Topics Cogn. Sci.* **14**, 93–110 (2022).

44. D. U. Wulff, S. Aeschbach, S. De Deyne, R. Mata, A data set to linking large-scale, individual semantic networks and cognitive performance. *PsyArXiv* (2021).

45. S. Bhatia, Predicting risk perception: New insights from data science. *Manag. Sci.* **65**, 3800–3823 (2019).

46. J. L. Tsai, T. Sims, Y. Qu, E. Thomas, D. Jiang, H. H. Fung, Valuing excitement makes people look forward to old age less and dread it more. *Psychol. Aging* **33**, 975–992 (2018).

47. M. K. Depping, A. M. Freund, Normal aging and decision making: The role of motivation. *Hum. Dev.* **54**, 349–367 (2011).

48. D. U. Wulff, T. Hills, R. Mata, Structural differences in the semantic networks of younger and older adults (2018); https://doi.org/10.31234/osf.io/s73dp.

49. M. Ramscar, C. C. Sun, P. Hendrix, H. Baayen, The mismeasurement of mind: Life-span changes in paired-associate-learning scores reflect the "cost" of learning, not cognitive decline. *Psychol. Sci.* **28**, 1171–1179 (2017).

50. D. U. Wulff, S. De Deyne, M. N. Jones, R. Mata, New perspectives on the aging Lexicon. *Trends Cogn. Sci.* **23**, 686–698 (2019).

51. R. Mata, R. Frey, D. Richter, J. Schupp, R. Hertwig, Risk preference: A view from psychology, *J. Econ. Perspect.* **32**, 155–172 (2018).

52. R. Frey, A. Pedroni, R. Mata, J. Rieskamp, R. Hertwig, Risk preference shares the psychometric structure of major psychological traits. *Sci. Adv.* **3**, e1701381 (2017).

53. R. Hertwig, D. U. Wulff, R. Mata, Three gaps and what they may mean for risk preference. *Philos. Trans. R. Soc. B* **374**, 20180140 (2019).

54. D. L. Nelson, C. L. McEvoy, S. Dennis, What is free association and what does it measure? *Mem. Cognit.* **28**, 887–899 (2000).

55. C. Zhao, S. Sahni, String correction using the damerau-levenshtein distance. *BMC Bioinformatics* **20**, 1–28 (2019).

56. G. G. Wagner, J. R. Frick, J. Schupp, "The German socio-economic panel study (SOEP): Scope, evolution and enhancements" (Technical Report SOEP papers on Multidisciplinary Panel Data Research, 2007).

57. A. Esuli, F. Sebastiani, SentiWordNet: A high-coverage lexical resource for opinion mining. *Evaluation* **17**, 26 (2007).

58. T. M. Fruchterman, E. M. Reingold, Graph drawing by force-directed placement. *Softw. Pract. Exp.* **21**, 1129–1164 (1991).

59. R. R. Bouckaert, E. Frank, Evaluating the replicability of significance tests for comparing learning algorithms, in *Proceedings of the Pacific-Asia Conference on Knowledge Discovery and Data Mining* (PAKDD, 2004), pp. 3–12.

60. A. Moors, J. De Houwer, D. Hermans, S. Wanmaker, K. Van Schie, A.-L. Van Harmelen, M. De Schryver, J. De Winne, M. Brysbaert, Norms of valence, arousal, dominance, and age of acquisition for 4,300 Dutch words. *Behav. Res. Methods* **45**, 169–177 (2013).

61. A. B. Warriner, V. Kuperman, M. Brysbaert, Norms of valence, arousal, and dominance for 13,915 English lemmas. *Behav. Res. Methods* **45**, 1191–1207 (2013).
